# Peripheral nerve function estimation by linear model of multi‐CMAP responses for surgical intervention in acoustic neuroma surgery

**DOI:** 10.14814/phy2.13495

**Published:** 2017-11-30

**Authors:** Dilok Puanhvuan, Sorayouth Chumnanvej, Yodchanan Wongsawat

**Affiliations:** ^1^ Department of Biomedical Engineering Faculty of Engineering Mahidol University Salaya Nakhon Pathom Thailand; ^2^ Surgery Department Faculty of Medicine Ramathibodi Hospital Mahidol University Bangkok Thailand

**Keywords:** Acoustic neuroma, CMAP, electrical stimulation, facial nerve preservation

## Abstract

Nerve function assessments are crucial for surgical intervention during acoustic neuroma surgery. Cranial nerves such as acoustic and facial nerves, can be possibly damaged during tumor dissection. Proper surgical intervention should prevent neurological deficit and achieve total tumor removal. Conventionally, nerve function is qualitatively evaluated by surgeon and neurologist. Facial nerves can be preserved by monitoring the compound muscle action potential (CMAP) response. The differences in the amplitude and latency of CMAP are used as indicators during surgical interventions. However, baseline CMAPs cannot be recorded in the presence of large acoustic tumors. This paper presents a new way of estimating nerve function. Instead of a single CMAP examination, multi‐CMAP responses are obtained from a train of varied stimulus intensities and these are applied a mathematical model. Shifts in the mathematical model parameters reflect changes in facial nerve function. In this study, experiments conducted in frog revealed that shifts in the linear model parameters were related to the level of induced nerve injury. Significant differences in the slope parameter of the linear model were found between each nerve condition. The identification of healthy and severed nerves via a support vector machine (SVM) corresponded to 94% accuracy. This classification criterion could be used with surgical intervention to prevent severed facial nerve palsy in acoustic neuroma surgery. The proposed method could be used to estimate nerve outcomes without prior information of a CMAP baseline.

## Introduction

An acoustic neuroma, also known as vestibular schwannoma, is a benign tumor originating from the vestibular nerve. It grows in the cerebellopontine angle area causing suppression and stretching of the brainstem and surrounding cranial nerves, including the trigeminal, cochlear, and facial nerves. Poor postoperative facial nerve outcomes such as Bell's palsy, can occur to the patient if the facial nerve is accidently damaged.

Preserving facial nerve function during acoustic neuroma surgery is critically important (Kartush et al. 1991; George Zouridakis 2001; O'Brien 2008; Moller 2011). Intraoperative monitoring systems (IOM) that can monitor various biological signals and apply external stimulation are standard in intracranial surgery. Monitoring continuous free‐running electromyography of facial muscles is often used. Mechanical irritation of facial nerve including touching, pulling, stretching, and compressing causes spikes and bursts in electromyogram (EMG) activity. Audible spikes and burst EMG can alert the surgeon of the facial nerve location (Prass and Lüders 1986; Johann et al. 2000; Kombos et al. 2000). Some studies have demonstrated that postoperative facial nerve function can be predicted by the duration of some specific burst EMG patterns (Prass and Lüders 1986; Johann et al. 2000). However, while the duration of burst EMG indicates mechanical irritation of the facial nerve, it does not correlate with short‐ or long‐term facial nerve function (Kombos et al. 2000). Burst EMG is also produced during coagulation and irrigation with saline. Some studies have predicted facial nerve outcomes from the tumor size (Nadol et al. 1992; Prakash et al. 1997). Nevertheless, postoperative facial nerve function depends on the surgeon's skill and the reliability of the intraoperative facial nerve monitoring system.

Evoked EMGs also known as compound muscle action potential (CMAP), evoked through a probe on the facial nerve proximal to the brainstem can help in estimating facial nerve function. Monitoring CMAP responses elicited from continuous stimulation is used for determining facial nerve location (Aage and Peter 1984). Some studies have demonstrated that facial nerve function is related to the stimulation threshold. A poor facial nerve outcome is observed for stimulus threshold ranges up to 0.84 mA (Silverstein et al. 1994; Colletti et al. 1997; Grayeli et al. 2005), but the stimulus threshold depends on the probe size and position. It has been found that CMAP amplitude reflects facial nerve function. A 50% reduction in CMAP amplitude has been used as a criterion for preventing severe postoperative facial palsy (Amano et al. 2011). This criterion has also been emphasized in transcranial electrical stimulation (TES) studies (Dong et al. 2005; Tokimura et al. 2014). However, the CMAP baseline generally cannot be recorded at the beginning of surgery in the presence of a large acoustic tumor (>2.5 cm diameter) because the tumor mass fully covers the operative area. Thus, the facial nerve may be injured before obtaining the baseline. TES is able to record the baseline, as it is noninvasive, but has adverse effects including tongue injury or seizures (Legatt 2002). High voltages applied through the electrode may also cause burns to the scalp. Peripheral nerves can be stimulated by certain electrical intensities. When the stimulus intensity is increased, the CMAP amplitude increases (McComas et al. 1971; Colletti et al. 1997; Tokimura et al. 2014). This property may be a useful basis for investigating facial nerve functional preservation.

Since single CMAP response is subject dependent and varied with probe position, decreasing in CAMP amplitude may not correlate to nerve function. Multi‐CMAP responses analysis could enhance nerve function prediction because nerve could be linearly modeled as mathematical equation from the input and output data. This study proposed a new modality of intraoperative facial nerve functional monitoring during acoustic neuroma surgery. Instead of a single CMAP response analysis, this study used multi‐CMAP responses elicited by varied stimulus intensities. The output data represented by the amplitude of multi‐CMAP responses and input data represented by the varied stimulus intensities were used for mathematical modeling. Shifts in the model parameters indicate changes in facial nerve function. A CMAP baseline may not be required in this proposed method because the model may self‐describe the remaining nerve function. Moreover, surgical interventions could be quantitatively prescribed based on changes in the nerve model parameters.

The sciatic nerve and gastrocnemius muscle of a frog represented the facial nerve and facial muscle in humans, respectively. Nerve injury, including compression and incision by the setup equipment, was used to imitate real operations in acoustic neuroma surgery.

## Materials and Methods

### Animal preparation

All applicable international, national, and institutional guidelines for the care and use of animals were followed. All procedures performed in studies involving animals were in accordance with the ethical standards of the animal care and use of the Mahidol University Animal Care and Use Commitees (COA No. MU‐ACUC 2014/001). Frogs are generally used in neural experiments because they have long sciatic nerves and large gastrocnemius muscles that can generate high‐amplitude signals by brief electrical pulses. Hence, in this study, the facial nerve and facial muscle were represented by a frog sciatic nerve and gastrocnemius muscle, respectively. Nerve‐muscle preparation in frog (East Asian bullfrog, 4 months) was performed in the following steps (Pal and Pal 2005; Elaine and Marieb 2010); (1) The frog was stunned by striking below the head to induce unconsciousness. (2) The unconscious frog was gently held to locate the depression point at the connection of the skull and the vertebral column. (3) A needle was firmly inserted to penetrate the skin, muscle and bone to the spinal cord. (4) The frog brain and the cord were damaged by manipulating the needle anterior into the skull and posterior into the spinal cord. This process is termed “pithing.” The frog remains alive but losses voluntary movement and other reflexes. (5) The skin was cut and removed from the middle trunk of the frog. (6) The urostyle was removed by cutting at pelvic girdle. (7) The sciatic plexus was identified and isolated. (8) The muscle was separated to expose the sciatic nerve at the thigh. (9) The gastrocnemius tendon was separated, cut, and tied. (10) Other muscle was removed and the tibia and femur were cut close to the knee joint. (11) The prepared nerve‐muscle was kept in a container containing Ringer solution (0.6% NaCl, 0.014% KCl, 0.012% CaCl2, 0.02% NaHCO3, and 0.1% dextrose).

### Electrical nerve stimulation and signal acquisition

A Digitimer DS3 (Digitimer Ltd, Hertfordshire, UK) current controlled (CC) stimulator was used to apply a pulse of monophasic electrical current to the sciatic nerve. The CC stimulator has four current ranges allowing precise control of output current between 2 *μ*A and 32 mA with adjustable pulse widths in the range of 10 *μ*sec–1000 msec. Current pulses of the desired intensity and pulse width were delivered to the sciatic nerve through a side‐by‐side bipolar stimulating probe (Cadwell Corp, Kennewick, WA). This pulse was controlled by a trigger signal from the microcontroller in the trigger unit. The pulse initiated action potentials (APs) in the sciatic nerve, which induce muscle twitching. Muscle contractions cause the generation of a compound muscle action potential (CMAP) captured by a twisted pair of subdermal needle electrodes (SGM Medical, Split, Croatia). Single subdermal needle electrode (SGM Medical, Split, Croatia) was used as a ground electrode and inserted at the gastrocnemius tendon. The CMAP signals were amplified using a BIOPAC MP100 amplifier (BIOPAC systems Inc). The gain and sampling frequency were 1000 and 2 kHz, respectively. The trigger signal was also acquired by the digital input of the BIOPAC MP100. EMG signals containing CMAP responses and trigger pulses were continuously monitored and recorded by AcqKnowledge 3.9.1 software (BIOPAC systems Inc). CMAP responses occur in EMG signals at some latency after the trigger signal.

### Nerve damage tools

During acoustic neuroma surgery, the cranial nerves in operative area, such as facial and vestibular nerves, can be damaged by starching, pulling, compressing, or incision. This study imitated nerve damage by manipulating the nerve via compression and incision.

### Nerve compression condition

**Figure 1:**
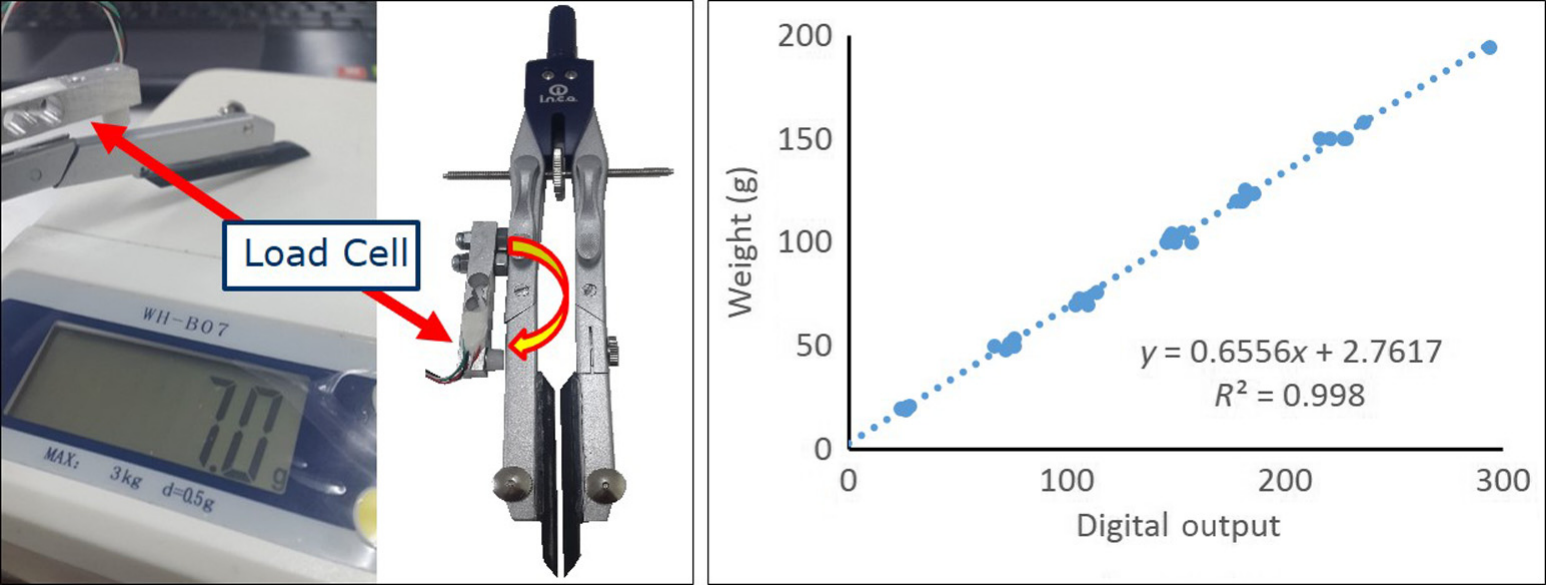
Nerve compression tool (left) and calibration results (right).

Nerve compression tools were built by installing a load cell (1 kg) to compasses (Fig. 1 (left)). Two customized 5‐mm‐wide acrylic plates were attached to each tip of the compasses. These plates could touch and compress the nerve, causing a repulsive force to the load cell. The analog output from the load cell was amplified and digitized by the analog front‐end (AFE) chip ADS1115 (Texas Instrument, Dallas, TX) where the gain setting and sampling frequency were eight times and 10 Hz, respectively. The ADS1115 was interfaced with a microcontroller (Arduino Uno) through an I2C bus. The nerve compression tool was calibrated by applying varied forces (grams) to the acrylic plate. The compass tip equipped with the load cell was held by an adjustable gripper. This gripper could gradual move the compasses tip toward the scales (0.5 g resolution, 3 kg maximum, Fig. 1 (left)).

Figure 1 (right) shows scatter plot of the applied weights read by the scales and the digital value reads by the microcontroller. Linearity was found between the input and output, hence, compression force at the nerve could be calculated by,

(1) y=0.6556x+2.7617

where *y* is the force at the nerve (gram), and *x* is digital value read from the load cell by the microcontroller.

### Nerve incision condition

This study used 1‐mm‐thin‐footage cervical Kerrison Rongeurs (Symmetry Surgical, Nashville, TN) for producing a nerve incision. Varied lateral incision depths by the cervical Kerrison Rongeurs represented different nerve injury levels.

### Experimental procedure

A pilot study was conducted to determine the nerve compression and incision level. Two sciatic nerves were used, one for nerve compression and one for nerve incision studies. The motor threshold (MT), the minimum stimulus intensity that could induce muscle twisting, was determined. Maximum compression (*Co*_max_) and incision (*In*_max_) levels were introduced when the CMAP response of 140%MT disappeared. The maximum injury level would be divided into three portions. Three levels of nerve damage by mechanical compression and incision were defined as Co_1_ (*Co*_max_/3), Co_2_ (Co_max_/2), Co_3_ (Co_max_), and In_1_ (In_max_/3), In_2_, (In_max_/2) In_3_ (In_max_), respectively.

Sixteen sciatic nerves were included in this study. Ten underwent nerve compression tasks and another six underwent nerve incision tasks. Both tasks were conducted as follows sequence:

1. Find the motor threshold (MT) by gradually increasing the current intensity until muscle twisting is observed.
2. Apply 4 trains of varied current intensities $P_{1-5}$ to obtain grand average CMAP responses (${\text{CMAP}}_{1-5}^{0}$).
  - Note, that P_1_ ≈ MT, P_2_ ≈ 110%MT, P_3_ ≈ 120%MT, P_4_ ≈ 130%MT, and P_5_ ≈ 140%MT, the pulse duration was 500 *μ*sec, and each pulse had approximately 1 sec interstimulus interval with approximately 5 sec between each pulse train.
3. Manipulate the nerve to the first damage level Co_1_ in the case of the nerve compression task or In_1_ in the case of the nerve incision task.
4. Apply 4 trains of varied current intensity stimulus $P_{1-5}$ to obtain grand average CMAP responses (${\text{CMAP}}_{1-5}^{1}$)
5. Manipulate the nerve to the second damage level Co_2_ in the case of nerve compression task or In_2_ in the case of the nerve incision task.
6. Apply 4 trains of varied current intensity stimulus $P_{1-5}$ to obtain grand average CMAP responses (${\text{CMAP}}_{1-5}^{2}$)
7. Manipulate the nerve to the third damage level Co_3_ in the case of the nerve compression task or In_3_ in the case of the nerve incision task.
8. Apply 4 trains of varied current intensity stimulus $P_{1-5}$ to obtain grand average CMAP responses (${\text{CMAP}}_{1-5}^{3}$)

The recorded EMGs containing the CMAP responses and trigger signals acquired by AcqKnowledge 3.9.1 software were exported as a .*mat* file for further analysis in MATLAB software (MathWorks Inc., Natick, MA). CMAP responses were segmented in the range from −5 to 20 msec according to the trigger pulse. These CMAP responses of each session were averaged from four stimuli trains as ${\text{CMAP}}_{1-5}^{x}$ according to the stimulus intensities ($P_{1-5}$) where *x* was the nerve conditions (initial (${\text{CMAP}}_{1-5}^{0}$), damage1 (${\text{CMAP}}_{1-5}^{1}$), damage2 (${\text{CMAP}}_{1-5}^{2}$), and damage3 (${\text{CMAP}}_{1-5}^{3}$) conditions).

### Nerve modeling

**Figure 2:**
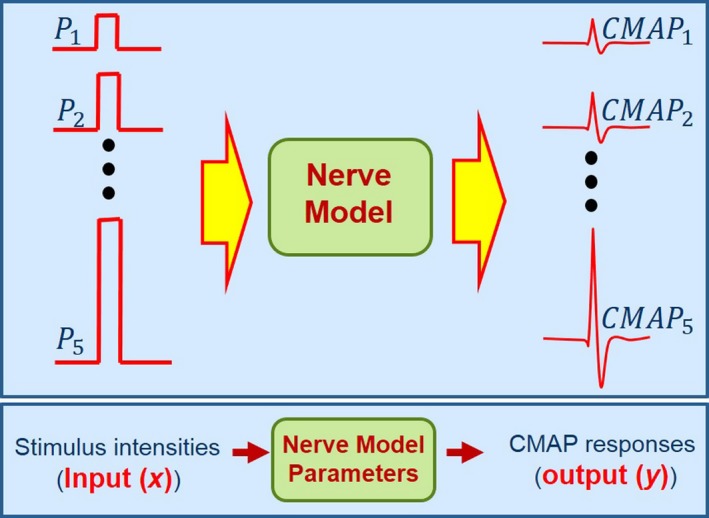
Block diagram of nerve modeling.

From explicit nerve‐muscles stimulation, motor output represented by CMAP responses increases in accordance with stimulus intensity (McComas et al. 1971; Colletti et al. 1997; Tokimura et al. 2014). Greater stimulus intensities allow more electrical current to penetrate through the axon in the nerve trunk, causing motor recruitment (Fig. 2). This study represented the nerve as a simple linear model denoted by (*y  =  ax + b*) where *x* was the varied stimulus intensity, and *y* was the CMAP response. The linear equation parameters *a* and *b* represent nerve model parameters that may vary depending on nerve function. When the nerve truck was injured by surgical manipulation such as pulling, starching, compressing, or incision, the output pattern of CMAP responses may be altered, causing the parameters (*a, b*) to change. Therefore, shifts in the parameters may represent indices for quantitative surgical intervention procedures.

Input data *x[n]* for nerve modeling could be determined by normalization based on the percentage of varied stimulus intensities $P_{1-5}$ according to the following equation:

(2) $$x\left[n\right]=\left(\frac{P_{n}-P_{1}}{P_{5}}\right)\times 100$$

where *n* was the index of stimulus intensities from 1 to 5.

The peak‐to‐peak voltage (*V*_pp._) of CMAP responses in the range from 1 to 15 msec after the stimulus onset was used as an output feature. The output data *y* ^*x*^ *[n]* of each session were determined by normalization based on the baseline (initial condition: ${\text{CMAP}}_{5}^{0}$) according to the following equation:

(3) $$y^{x}\left[n\right]=\left(\frac{{\text{CMAP}}_{n}^{x}-{\text{CMAP}}_{1}^{x}}{{\text{CMAP}}_{5}^{0}}\right)\times 100$$

,where *x* represents the nerve conditions (baseline (*x *=* *0), damage level 1 (*x *=* *1), damage level 2 (*x *=* *2), and damage level 3 (*x *=* *3) conditions), *and n* is the index of the stimulus intensities from 1 to 5.

Linear equation parameters (*a, b*) of each nerve condition were calculated by linear least square solution (Douglas et al. 2012) as shown in Equation (4).

(4) $$\theta ={\left(X^{T}X\right)}^{-1}X^{T}y$$

where $\theta =\left[\begin{array}{c} a\\ b \end{array}\right]$, $X=\left[\begin{array}{cc} 1 & P_{1}\\ : & :\\ 1 & P_{5} \end{array}\right]$, $y=\left[\begin{array}{c} {\text{CMAP}}_{1}^{x}\\ :\\ {\text{CMAP}}_{5}^{x} \end{array}\right]$

## Results

**Figure 3:**
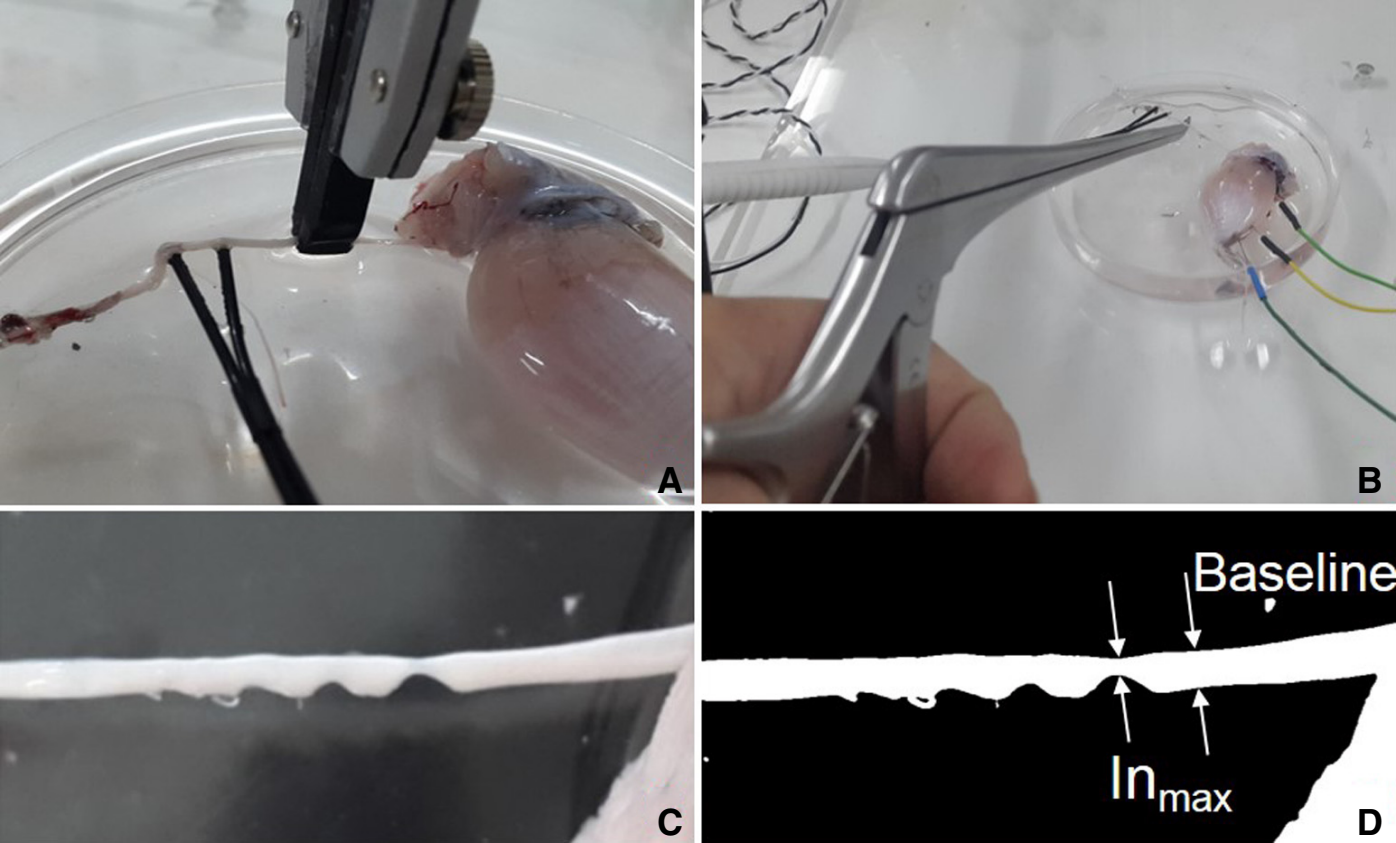
Inducing nerve injury to a frog's sciatic nerve by compression (*A*) and incision (*B*) tools. *C* is frog's sciatic nerve after incision by the cervical Kerrison Rongeurs and *D* is a binary image of *C*.

Figure 3 shows nerve damage by mechanical compression (Fig. 3(*A*)) and incision (Fig. 3(*B*)). The results of the pilot study showed that the maximum compression (Co_max_) that could disable the CMAP response at 140%MT was approximately 90 g. Hence, three injury levels for the nerve compression task were 30, 60, and 90 g for Co_1_, Co_2_, and Co_3_, respectively.

Figure 3(*C*) shows nerve incision by the cervical Kerrison Rongeurs. The lateral depth incision was varied by the surgeon. The maximum incision (In_max_) could be found by increasing the lateral incision depth until the CMAP response at 140%MT disappeared. Figure 3(*C*) was converted to a binary image, as shown in Figure 3(*D*). In_max_ was calculated as percent different between the remaining white pixels at the maximum incision area compared to baseline. The In_max_ was 63.2%. Therefore, the three injury levels for the nerve incision experiment were approximately 20%, 40%, and 60% for In_1_, In_2_, and In_3_, respectively.

After the experiment was completed for all sciatic nerves, injury levels (damage1, 2, and 3) from the compression and incision tasks were considered. Inducing a certain incision level was difficult. The damage levels induced by cervical Kerrision Rongeurs on six sciatic nerves were 21.87 ± 1.60 (SE) %, 36.15 ± 3.12 (SE) %, and 55.44 ± 5.04 (SE) % for In_1_, In_2_, and In_3_, respectively. The damage levels induced by the nerve compression tools on 10 sciatic nerves were 30, 60, and 90 g for Co_1_, Co_2_, and Co_3_, respectively.

**Figure 4:**
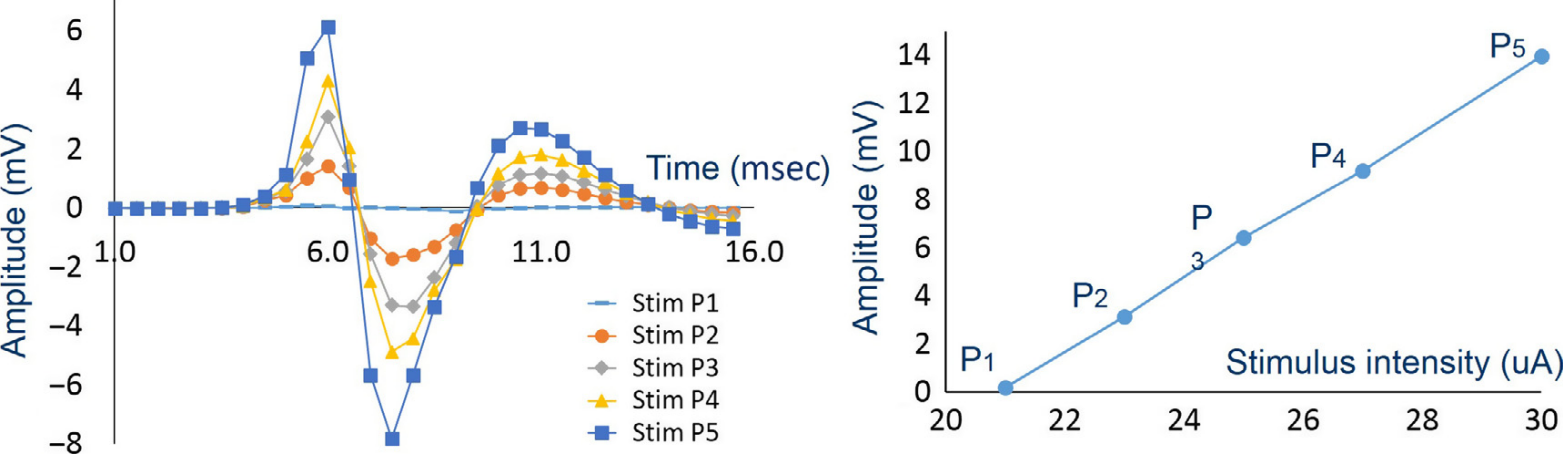
CMAP responses elicited by varied stimulus intensities (P_1_ (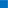), P_1_ (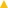)_*,*_ P_1_ (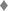)_*,*_ P_1_ (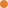), and P_1_ (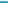)) during the initial condition in subject 1 (left) and relationship between voltage peak‐peak (*V*_pp_) of CMAP responses (${\text{CMAP}}_{5}^{1}$) and varied stimulus intensities (P_1‐5_) during the initial condition in subject 1

Figure 4 (left) shows example CMAP responses elicited by varied stimulus intensities (P_1_ (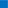), P_1_ (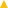)_*,*_ P_1_ (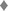)_*,*_ P_1_ (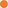), and P_1_ (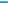)) during the initial condition (${\text{CMAP}}_{5}^{0}$) of subject 1. As the stimulus intensity was increased, the amplitude of the CMAP response increased.

The *V*_pp_ of the grand average CMAP response was determined. Figure 4 (right) shows an example plot between *V*_pp_ of the CMAP responses (${\text{CMAP}}_{5}^{0}$) and the varied stimulus intensities (P_1‐5_) during initial condition in subject 1. The results showed that the *V*_pp_ of the CMAP response was linearly correlated with the applied stimulus intensity. Hence, modeling the proposed nerve model as a linear equation was suitable in this study.

**Figure 5:**
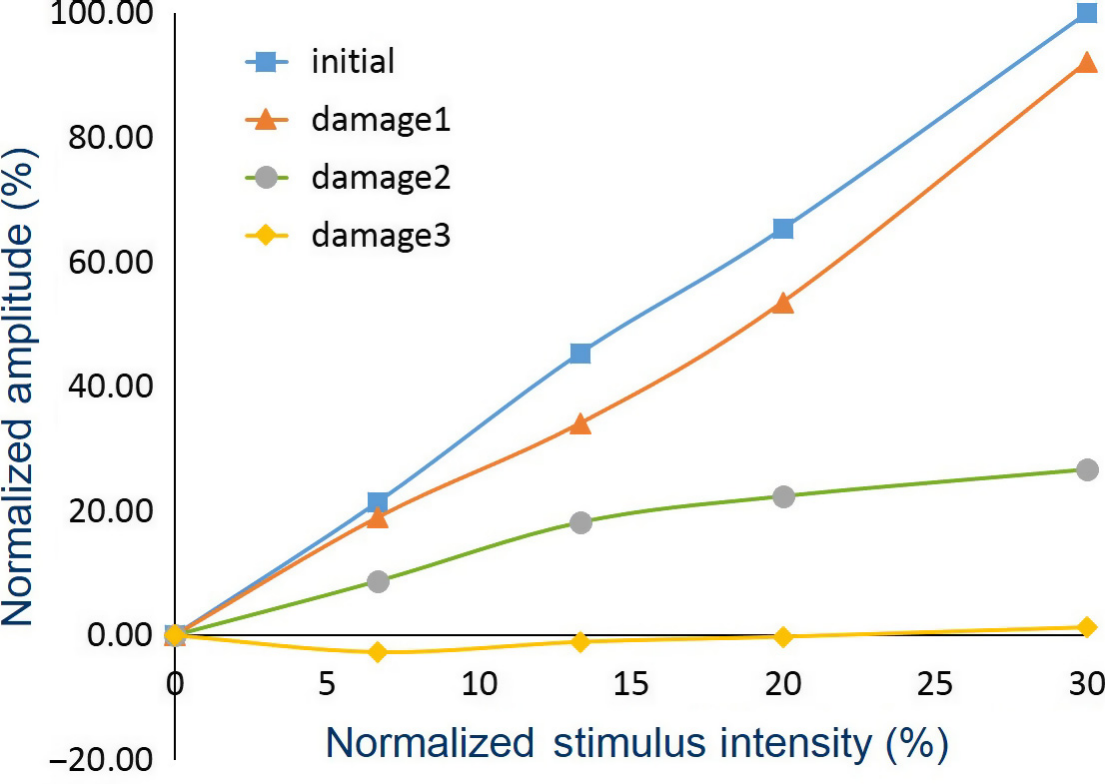
Relationship between the normalized *V*_pp_ of the CMAP responses (${\text{CMAP}}_{1-5}^{x}$) and the stimulus intensities (P_1‐5_) of all nerve conditions (initial (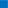), damage1 (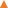), damage2 (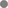), and damage3 (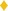) conditions) during the experiment of subject 1.

The *V*_pp_ of the CMAP responses and stimulus intensities of all nerve conditions in all subjects were normalized by Equations (2) and (3), respectively. Figure 5 shows an example plot between the normalized *V*_pp_ (%) and the stimulus intensities (%) for all nerve conditions (initial (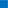) and damage 1 (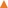), damage 2 (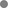), and damage 3 (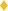) conditions) during the experiment of subject 1. The results revealed that the linear relationship of *V*_pp_ and stimulus intensity was shifted for each nerve condition. The initial condition graph corresponded to the highest slope. When the nerve was damaged, the slope decreased according to the severity of the injury level.

**Figure 6:**
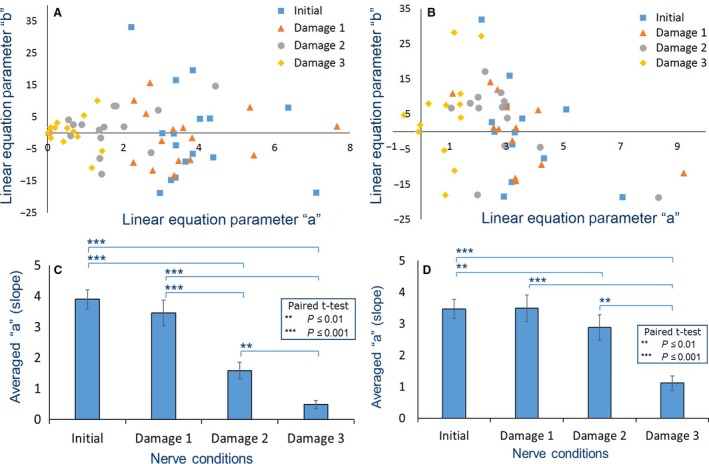
Scatter plot of linear equation parameters *a* and *b* by baseline‐based normalization (*A*) and per‐trial‐based normalization (*B*) of initial (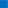) and damage1 (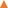), damage2 (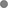), and damage3 (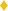) conditions of all sciatic nerves and bar graph of the mean (*n *=* *16) linear equation parameter *a* by baseline‐based normalization (*C*) and per‐trial‐based normalization (*D*) of each nerve condition.

The normalized stimulus intensity represented by *x[n]* and the *V*_pp_ of the CMAP response represented by *y[n]* of each nerve condition for each sciatic nerve were input into Equation (4). The linear equation parameters (*a* and *b*) were calculated and plotted as shown in Figure 6 *(A)*. The plot of initial (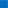) and mild injury (damage1;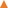) conditions was clearly distinguishable from severe injury (damage3;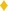).

Paired t‐tests were used for statistical analysis. Significant differences were found for parameter *a* of each nerve condition, as shown in Figure 6(*C*). The initial and damage1 conditions were significantly different from the damage2 and damage3 conditions at *P* ≤ 0.001. Significant different at *P* ≤ 0.01 were found between the damage2 and damage3 conditions. There was no significant difference for any nerve condition in linear equation parameter *b*.

The results show the possibility of nerve function prediction by considering shifts in the linear equation parameters. Instead of nerve function validation with a single CMAP response, the proposed multi‐CMAP responses would be more reliable. Nevertheless, this result was determined by normalization based on prior knowledge of the initial condition (baseline). In real cases, the nerve generally cannot be observed within a large acoustic neuroma, and the nerve may be injured due to surgical manipulation. Hence, the baseline may not truly represent the initial condition. Thus, nerve function estimation would not be accurate in this case.

Further analysis was conducted for nerve function estimation without the CMAP baseline. Each trial was self‐normalized within a particular trial (${\text{CMAP}}_{5}^{x}$) by Equation (5),

(5) $$y_{\mathrm{new}}^{x}\left[n\right]=\left(\frac{{\text{CMAP}}_{n}^{x}-{\text{CMAP}}_{1}^{x}}{{\text{CMAP}}_{5}^{x}}\right)\times 100$$

where *x* is nerve condition, and *n* is the index of stimulus intensities from 1 to 5.

The new normalized *V*_*pp*_ of CMAP response represented by $y_{\mathrm{New}}^{x}\left[n\right]$ and the stimulus intensity represented by *x[n]* of each nerve condition were substituted into Equation (4). Figure 6 (*B*)shows a scatter plot of the linear equation parameters (*a* and *b*) for “per‐trial” based normalization in the initial (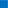) and damage1 (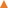), damage2 (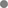), and damage3 (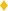) conditions. The results revealed similar distributions between the baseline‐based normalization (Fig. 6(*A*)) and per‐trial‐based normalization Figure 6(*C*). Statistical analysis by paired t‐tests demonstrated that significant differences between each nerve condition could be found in the linear equation parameter *a* as shown in Figure 6(*D*). Initial and damage1 conditions were significantly different (*P*‐value ≤ 0.001) to damage3 condition. Significant differences at *P* ≤ 0.01 were found between the initial and damage2 to damage3 conditions.

**Figure 7:**
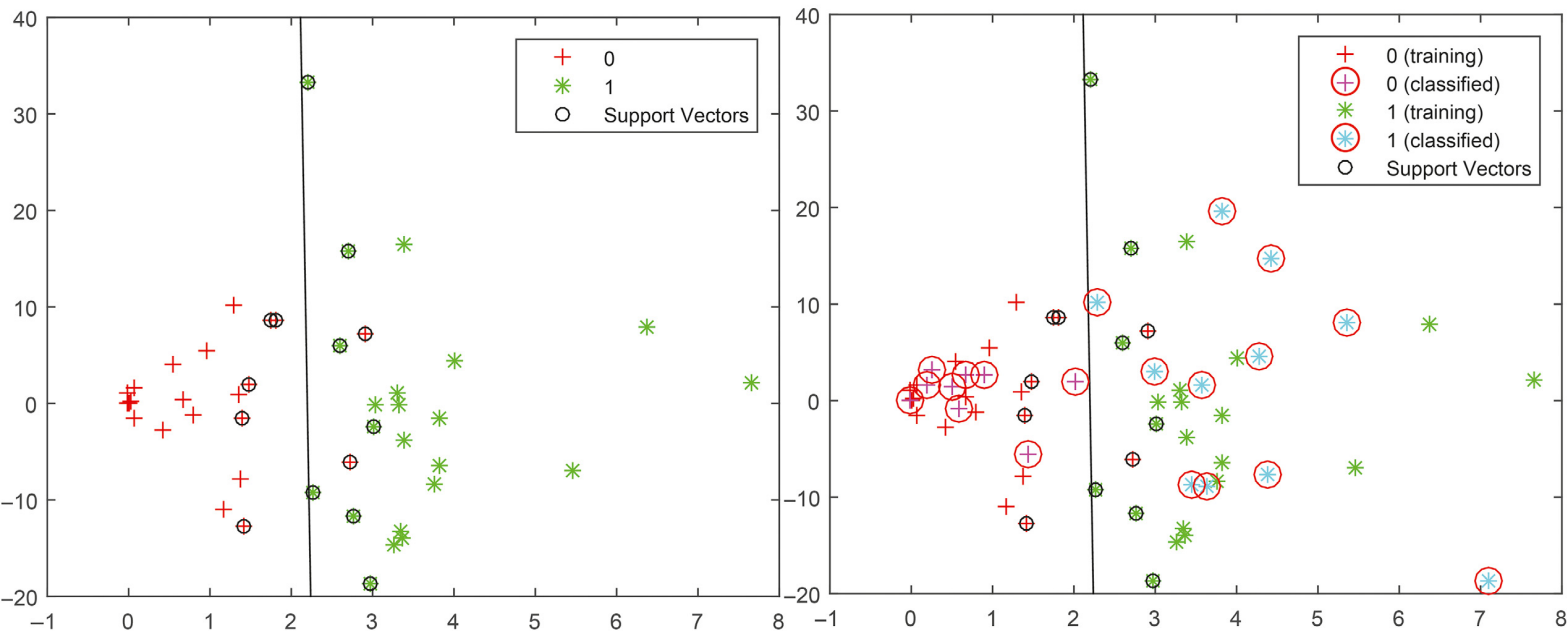
The line separating the training set of healthy (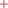) and injured (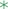) calculated by the SVM (left) and classification the results of the test set data (in the circle: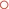) of healthy (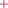) and injured (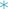) (right).

Changes in linear equation parameter could be used for estimating nerve functions. Likewise, a surgical intervention index could be determined from these parameters. In this study, the dataset of the initial and damage1 condition (*n *=* *32) were considered as healthy nerve condition, whereas the data for the damage2 and damage3 conditions were considered as injured condition (*n *=* *32). These two sets of parameters categorized as healthy and injured were used to train a classifier. A supervised learning support vector machine (SVM) classifier was employed in this study (Suykens and Vandewalle 1999; Chang and Lin 2011). The MATLAB function “*svmtran(data, group)*” was used to separate the healthy and injured datasets. All data points (64 points) of baseline‐based normalization data were random and separated to 70% and 30% for the training set (44 points) and test set (20 points), respectively. Figure 7 (left) shows that the training set data (healthy: 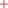
and injured: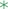) was separated by a black line calculated from the support vectors.

Figure 7 (right) show the classification results of the test set data (in the circle: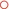) of healthy (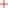) and injured (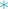) points. The classification accuracy was 95%.

The SVM classifier trained by the training set via baseline‐based normalization was used for classifying the per‐trial‐based normalization dataset. The results revealed that the classification accuracy of the initial (16 data points), damage1 (16 data points), damage2 (16 data points), and damage3 (16 data points) conditions were 93.8%, 93.8%, 31.3%, and 81.3%, respectively.

## Discussion

Generally, postoperative facial nerve function outcome is assessed by the House Brackmann (HB) grading system (Nadol et al. 1992; Silverstein et al. 1994; Colletti et al. 1997; Prakash et al. 1997; Kombos et al. 2000; Dong et al. 2005; Amano et al. 2011; Tokimura et al. 2014). The HB grading system ranges from grades of I to VI, where grade I refers to normal function, and grade VI indicates complete paralysis. The amplitude of CMAP responses is related to nerve function (Colletti et al. 1997; Dong et al. 2005; Amano et al. 2011; Tokimura et al. 2014), and poor postoperative nerve outcomes are observed with an absence of CMAP responses (Colletti et al. 1997; Dong et al. 2005; Tokimura et al. 2014). This study induced nerve injury using nerve compression and incision tools. The absence of CMAP responses at a stimulus intensity of 140%MT during maximum damage level would correspond to an HB grade of V or VI. Nerve compression levels at *Co*_*1*_, *Co*_*2*_, and *Co*_*3*_ were controlled by adjusting the modified compasses angle, while nerve incision levels depended on surgeon. However, the nerve incisions at each damage level could be assessed. Only small variations were observed in *In*_*1*_, *In*_*2*_, and *In*_*3*_, which corresponded to 21.9 ± 1.6 (SE) %, 36.2 ± 3.2 (SE) %, and 55.4 ± 5.0 (SE) %, respectively.

The MT varied in different sciatic nerves. The CMAP amplitude increased when the stimulus intensity was increased, as more axons become activated by the greater electric field that penetrates the nerve trunk from the increased stimulus intensity (McComas et al. 1971). The amplitude of the grand averaged CMAP response had a linear relationship with the stimulus intensity. Hence, the proposed linear model was applicable for extracting the nerve function features. This information confirms our preliminary report that a nerve model may be represented by a linear equation and a linear model parameter may reflect nerve function (Puanhvuan et al. 2013). The latency and morphology of the CMAP responses of the frog nerve‐muscle preparations may differ from CMAP responses of facial nerve‐muscles in human. However, this dissimilarity could be neglected by the proposed normalization method. Normalization could be done based on information of the initial condition (baseline‐based normalization) or based on each trial (per‐trial‐based normalization). Existing studies have demonstrated that a 50% difference in observed CMAPs compared to baseline CMAPs can be used as a criterion to prevent nerve palsy (Dong et al. 2005; Amano et al. 2011; Tokimura et al. 2014). Similar to this nerve function preservation method that requires a CMAP baseline, baseline‐based normalization requires prior knowledge of the CMAP response at the initial state. The distribution in the scatter plot of the calculated linear equation parameters (*a* and *b*) exhibited a clear separation between the initial and damage2 to damage3 conditions. Significant differences in the slope of equation (parameter *a*) were found for each nerve condition. When the sciatic nerve was injured, the slope of the linear relationship was decreased in accordance with the severity of nerve injury. There was no significant different found in parameter *b* due to normalization process. Linear equation parameter b, offset on *y*‐axis, should be ideally zeros since ${\text{CMAP}}_{1}^{x}$ (MT, CMAP according to *P*_*1*_) and *P*_*1*_ were normalized as 0%. Variation in *b* was presented due to some data point in ${\text{CMAP}}_{1-5}^{x}$ was outlier. The decreasing CMAP amplitude might be caused by a loss of remaining excitable axons due to the mechanical compression and incision processes. In this study, type of injury cannot be defined by the obtained results. Mechanical compression and incision conditions generates similar acute injury outcome. Nonetheless, nerve injury levels could be revealed by linear model of multi‐CMAP responses. Nerve function estimation by multi‐CMAP responses would be more reliable than a single CMAP response analysis.

Similarities in the distribution of the baseline‐based normalization data were found in the per‐trial‐based normalization data. Significant differences were also found in each nerve condition on the linear equation slope (parameter a). Hence, it is feasible to estimate nerve function without baseline information. This method could be applied to any application that requires nerve function preservation following an acute nerve injury.

Surgical intervention is critically important for preventing postoperative neurological deficits. Multiclass classification is feasible for classifying the four damage levels (initial, damage1, damage2, and damage3). The surgeon could monitor and aware of poor postoperative outcome according to the degree of injury. However, the obtained results of linear equation parameter were not clearly separated in every conditions, for example; initial and damage1, and damage2 and damage3. Further development on classification should be investigated. Hence, in this study, the surgical intervention index used for indicating healthy or injured nerve was considered. Initial and light injury (damage1) conditions were defined as healthy data. To prevent poor nerve function outcomes, severe injury in the damage2 and damage3 conditions was considered injured data. The line separating these two datasets was calculated by the SVM. The training set was induced 70% (*n *=* *44) of the baseline‐based normalization data. The classification accuracy of the test set (another 30% (*n *=* *20) of baseline‐based normalization) was 95%. The separation line calculated from the training set of the baseline‐based normalization data was used for classifying the per‐trial‐based normalization data. A 93.8% accuracy was achieved in the initial and damage1 conditions. The damage2 condition showed the lowest accuracy, which might be due to the variation in inducing this type of nerve injury.

The proposed surgical intervention method, which was classified by a SVM would benefit facial nerve preservation in acoustic neuroma surgery. A quantitative indication of healthy or injured conditions from the multi‐CMAP response would be superior to the convention method based on analysis of a single CMAP (Silverstein et al. 1994; Colletti et al. 1997; Dong et al. 2005; Amano et al. 2011; Tokimura et al. 2014). Facial nerve preservation reported by existing studies required baseline CMAP for calculating percent different (Dong et al. 2005; Amano et al. 2011; Tokimura et al. 2014).The proposed method does not require a baseline, as the multi‐CMAP amplitude can be self‐normalized and, thus, self‐describes the nature of the nerve. Total tumor removal may be achieved as the remaining nerve function is quantitatively revealed to the surgeon. The results of this animal study feasibly predicted nerve function during acoustic neuroma surgery.

## Conflict of Interest

All authors have received research grants from Mahidol University. Wongsawat Y. and Chumnanvej S. have been employed by Mahidol University. Puanhvuan D. is the Ph.D. candidate under supervision of Wongsawat Y. at the Faculty of Engineering, Mahidol University. The authors declare that they have no conflict of interest.
